# The impact of age on efficacy and safety of disease modifying treatment—insights from the Austrian Multiple Sclerosis Treatment Registry

**DOI:** 10.1007/s00415-026-13885-z

**Published:** 2026-06-12

**Authors:** Michael Guger, Christian Enzinger, Bettina Heschl, Franziska Di Pauli, Christiane Gradl, Stefan Kalcher, Erich Kvas, Thomas Berger, the Austrian MS Treatment Registry (AMSTR)

**Affiliations:** 1 Department of Neurology, Pyhrn-Eisenwurzen Hospital Steyr, Sierninger Straße 170, 4400 Steyr, Austria; 2 Medical Faculty, Johannes Kepler University Linz, Linz, Austria; 3 Department of Neurology, Medical University of Graz, Graz, Austria; 4 Clinical Department of Neurology, Medical University of Innsbruck, Innsbruck, Austria; 5 Department of Neurology, Medical University of St. Pölten, St. Pölten, Austria; 6 Hermesoft, Data Management, Graz, Austria; 7 Hermesoft, Statistics, Graz, Austria; 8 Department of Neurology, Medical University of Vienna, Vienna, Austria; 9 Comprehensive Center for Clinical Neurosciences & Mental Health, Medical University of Vienna, Vienna, Austria

**Keywords:** Age, Efficacy, Multiple sclerosis, Real-world, Registry, Safety

## Abstract

**Introduction:**

Efficacy and safety considerations in the treatment of multiple sclerosis (MS) change over a person's lifetime due to immunosenescence and the increasing prevalence of comorbidities with advancing age.

**Objectives/aims:**

To evaluate the overall efficacy and safety of disease-modifying therapies in MS patients across different age groups using prospectively collected real-world data from a nationwide observational cohort.

**Methods:**

We included patients from the Austrian MS Treatment Registry (AMSTR) who were receiving treatment with Alemtuzumab, Cladribine, Dimethyl fumarate, Fingolimod, Natalizumab, Ocrelizumab, Ofatumumab, Ozanimod, Ponesimod, Siponimod, and Teriflunomide for at least 12 months as of March 2025. Patients were categorized into two age groups: < 50 years (n = 1,459) and ≥ 50 years (n = 658).

A generalized linear model (GLM) and Cox proportional hazards models were applied to assess treatment effects on annualized relapse rates (ARR) and changes in the Expanded Disability Status Scale (EDSS), including both progression and improvement.

**Results:**

Over a mean treatment duration of 6.0 years (younger cohort) and 7.8 years (older cohort), the estimated mean ARR was 0.12 and 0.09, respectively (p < 0.001). Cox regression analysis of time to first relapse yielded a hazard ratio (HR) of 1.34 (95% CI 1.12–1.60, p = 0.001), indicating a significantly higher relapse risk for the younger cohort.

For sustained EDSS progression at 12 and 24 weeks, the corresponding HRs were 0.76 (95% CI 0.64–0.912, p = 0.003) and 0.70 (95% CI 0.58–0.85, p < 0.001), respectively, demonstrating a higher risk of disability progression in the older cohort.

**Conclusion:**

Our findings indicate that approximately one-third of treated MS patients in our registry are aged 50 years or older, underscoring the substantial presence of older patients in contemporary MS treatment cohorts. Furthermore, efficacy outcomes, including ARR and EDSS progression, differed significantly between younger and older age groups. These results suggest that both disease activity and chronological age are primary determinants of treatment outcomes.

## Introduction

To date, the focus of multiple sclerosis (MS) treatment has predominantly been on younger patients, for whom early and effective therapy is recommended once a definite diagnosis has been established. In recent years, however, the age distribution of affected individuals has increasingly shifted toward older age groups—particularly between 50 and 65 years—as demonstrated by various data [12, 17, 31].

In this age group, specific factors must be taken into account when making treatment decisions: immunosenescence, a higher prevalence of comorbidities, and increased polypharmacy. Immunosenescence is associated with an increased risk of infections and more severe disease courses, as well as reduced responses to vaccinations [19, 20].

In addition, particularly psychiatric and cardiovascular comorbidities may limit therapeutic options [27]. The clinical relevance of comorbidities has been demonstrated in a nationwide Austrian analysis, where Zinganell et al. showed that psychiatric comorbidities, dementia, and osteoporosis have a specific negative impact on disease course [34]. Furthermore, polypharmacy is prevalent among older MS patients and may increase the risk of drug-drug interactions [4].

Conversely, discontinuation of highly effective therapies frequently leads to recurrence of disease activity—both clinically and radiologically—even beyond the age of 50 years [3, 5, 18], underscoring the continued need for effective treatment in this population.

Most of pivotal clinical trials in MS included only patients up to the age of 55 years, with the exceptions of siponimod and tolebrutinib (up to 60 years), and ocrelizumab (up to 65 years) [10, 16]. Consequently, data on efficacy, tolerability and safety in older patients are derived primarily from observational studies.

Moderately effective disease-modifying therapies (mDMTs) [33] have demonstrated comparable efficacy and tolerability in both younger and older patients [2, 22]. However, analyses of dimethyl-fumarate (DMF) showed more frequent reductions in T-cell counts and higher rates of treatment discontinuation due to adverse events in older patients [22].

Regarding highly effective DMT (hDMTs), evidence also supports comparable efficacy across different age groups [6, 7, 16]. With cladribine, slightly increased rates of grade 3/4 lymphopenia with corresponding adverse effects were observed in patients older than 50 years [11]. In a retrospective analysis of highly effective therapies, the incidence of adverse events was comparable between patients younger than 50 years and those aged 50 years or older, with the exception of urinary tract infections [29].

Despite these observations, real world evidence comparing treatment outcomes across age groups remains limited. In this study, we therefore aimed to evaluate the overall efficacy and safety of DMTs in MS patients across different age groups using data form a nationwide observational cohort (Austrian MS Treatment Registry).

## Materials and methods

### Data collection

The Austrian MS Treatment Registry (AMSTR), established in 2006, serves as a platform for maintaining quality control and ensuring compliance with reimbursement regulations set by Austrian sick fund [13, 14]. The systematic registry enables to collect clinical data, to assess treatment indications and patient profiles, and to monitor drug safety in a real-world setting.

At the time of the AMSTR’s establishment, treatments with interferon-beta and glatiramer acetate were not included. The registry operates within a comprehensive network of approximately 100 MS centers across Austria. These centers consist of MS clinics within neurological departments and dedicated neurological medical offices that have been accredited by the Austrian Society of Neurology based on stringent quality criteria. Notably, the prescription of disease-modifying therapies (DMTs) for MS is exclusively reserved for these certified MS centers, ensuring consistent documentation and even distribution of treatment across Austria.

The AMSTR complies with Austrian bioethics laws and was approved by the Ethics Committee of the Medical University of Vienna (EC number 2096/2013).

The registry records anonymous baseline data, including the date of MS clinical onset, disease duration, relapses in the previous 12 months, EDSS (Expanded Disability Status Scale) scores, brain magnetic resonance imaging (MRI) activity, and prior DMT usage. Follow-up data, including relapses, EDSS progression, adverse events (AEs), and treatment changes or discontinuations, must be documented every 3–6 months. Due to the registry's structure and requirements, brain and spinal cord MRI data is collected only at baseline, prior to treatment initiation, and not during follow-up.

Each relapse must be confirmed by a neurologist at the MS center and documented within the registry. Documentation includes the date of relapse onset, EDSS score, and details regarding the use and dosage of intravenous methylprednisolone treatment.

In addition to being a mandatory requirement for reimbursement, the AMSTR features external and independent data monitoring. This process enhances data quality by improving the completeness, accuracy, and plausibility of the documented information, representing a unique quality standard of the AMSTR. Therefore, we did not identify any relevant missing data among the patients included in the AMSTR.

For the present study, we included patients from the AMSTR who were currently (most recent visit within the last 6 months) receiving treatment with Alemtuzumab (AZM), Cladribine (CLAD), Fingolimod (FTY), Natalizumab (NTZ), Ocrelizumab (OCR), Ofatumumab (OFA), Ozanimod (OZA), Ponesimod (PONE) and Siponimod (SIPO) (highly effective DMTs) or Dimethyl fumarate (DMF) and Teriflunomide (TERI) (moderately effective DMTs) as of March 2025 and had at least 12 months of follow-up observation. Patients were categorized into two age groups: < 50 years (n = 1459) and ≥ 50 years (n = 658).

### Outcome measures

We defined the annualized relapse rate (ARR) during treatment as the primary outcome measure. Secondary outcome measures included the time to first relapse, sustained disability progression or improvement, EDSS change and frequency of adverse events (AEs). Sustained disability progression was defined as an increase of at least 1.0 point on the EDSS for patients with a baseline EDSS score between 0 and 5.0, or an increase of at least 0.5 points for those with a baseline score greater than 5.5. Conversely, sustained disability improvement was defined as a decrease of 1.5 points in the EDSS for patients with a baseline score of 1.5, a decrease of at least 1.0 point for those with a baseline EDSS between 1.5 and 5.0, or a decrease of at least 0.5 points for those with a baseline score above 5.5.

These changes had to be confirmed after 12 or 24 weeks and maintained at the same level or greater until the last available EDSS score within the observation period. To evaluate these outcomes, one EDSS assessment was required at initiation and on average every three to six months, depending on the specific monitoring requirements of treatments in Austria.

Adverse events were reported according to MedDRA system organ classes.

### Statistical methods

All models included the following factors: age, sex, disease duration and EDSS score at baseline, relapse rate within the 12 months prior to treatment initiation, the presence of at least nine T2 hyperintense lesions and at least one gadolinium-enhancing (Gd-enhancing) T1 lesion on MRI at baseline and type of treatment (moderately or highly effective DMTs) at baseline.

All models were generated twice: Once with age as continuous variable for optimal usage of age information, and once with age as categorical grouping variable (< 50 years vs. ≥ 50 years) to investigate the effect of a discrete limit. With the age grouping variable it was possible to estimate ARR per age group from the model, and Kaplan Meier estimates could be compared between younger and older patients.

A generalized linear model (GLM) was used, with relapse count as a Poisson-distributed dependent variable and duration of treatment period as an offset variable, to identify factors influencing the annualized relapse rate (ARR).

Cox proportional hazards models were applied to identify factors affecting the time to first relapse, EDSS progression confirmed after 12 and 24 weeks, and EDSS regression confirmed after 12 and 24 weeks.

The proportional hazards assumption for the Cox models had been verified by non-significant deviations from the proportional hazards assumption for each covariate in the model using Schoenfeld residuals.

As statistical programmes, we used IBM SPSS Statistics for Windows, Version 24.0 (Armonk, NY: IBM Corp.), Stata Statistical Software, Release 17 (College Station, TX: StataCorp LLC.).

## Results

According to the predefined inclusion criteria, the cohort comprised 1,459 RRMS patients aged < 50 years and 658 patients aged ≥ 50 years at reference date. Mean age at the reference date was 38 and 56 years, while mean age at the time of diagnosis was 34 and 47 years, respectively. Further baseline characteristics at the reference date and prior to treatment initiation are summarized in Table 1.

**Table 1:** Patient characteristics at reference date and prior treatment start

			< 50 years N = 1459	≥ 50 years N = 658
Female		N	988	431
		%	67.7%	65.5%
Age	Mean		38	56.3
	SD		7.2	4.8
Duration of MS at reference date (years)*	Mean		4.3	9.8
	SD		5.2	8.0
EDSS at reference date	Mean		1.6	2.6
	SD median		1.3 1.5	1.5 2.5
Relapse rate within 12 months prior treatment start	Mean		1.3	1.1
	SD		1.0	1.0
≥ 9 T2 lesions	Yes	N	1156	591
		%	79.5%	90%
	No	N	298	66
		%	20.5%	10%
≥ 1 Gd-enhancing T1 lesion	Yes	N	736	285
		%	50.8%	44%
	No	N	713	362
		%	49.2%	56%
Previous Treatment	Yes	N	763	453
		%	52.3%	68.8%
	No	N	696	205
		%	47.7%	31.2%
Follow-up in years	Mean		6.0	7.8
	SD		3.9	4.5

*EDSS* expanded disability status scale, *Gd* gadolinium, *MS* multiple sclerosis, *SD* standard deviation

The most commonly used first-line therapies in both cohorts were DMF, NTZ and FTY (Table 2). In the younger cohort, 536 patients (37%) switched treatment, compared with 214 patients (33%) in the older cohort, most frequently from DMF, NTZ and FTY (Table 2).

**Table 2:** Initial and final treatment distribution

Treatment	< 50 years Initial treatment	< 50 years Final treatment	≥ 50 years Initial treatment	≥ 50 years Final treatment
AZM	8	7	1	1
CLAD	56	112	10	26
DMF	528	327	166	129
FTY	224	191	145	160
NTZ	289	228	143	71
OFA	75	240	10	41
OCR	79	150	46	73
OZA	72	89	15	28
PONE	14	41	8	13
SIPO	6	15	28	48
TERI	108	59	86	65

*AZM* alemtuzumab, *CLAD* cladribine, *DMF* dimethylfumarate, *FTY* fingolimod, *NTZ* natalizumab, *OCR* ocrelizumab, *OFA* ofatumumab, *OZA* ozanimod, *PONE* ponesimod, *SIPO* siponimod, *TERI* teriflunomide

Among younger patients, treatment switches were most commonly to OFA (n = 165), OCR (n = 79), NTZ (n = 69), CLAD (n = 62) and FTY (n = 54). In contrast, older patients most frequently switched to FTY (n = 60), OFA (n = 31), OCR (n = 29), SIPO (n = 21) and CLAD (n = 17).

To address the first study objective—comparing overall treatment efficacy across age groups—the estimated ARR was 0.12 (SD: 0.004) in younger patients and 0.09 (SD: 0.005) in older patients over a mean treatment duration of 6.0 years (SD: 3.9) and 7.8 years (SD: 4.5), respectively, yielding an incidence rate ratio (IRR) of 1.32 (95% CI 1.17–1.48; p < 0.001).

**Fig. 1:**
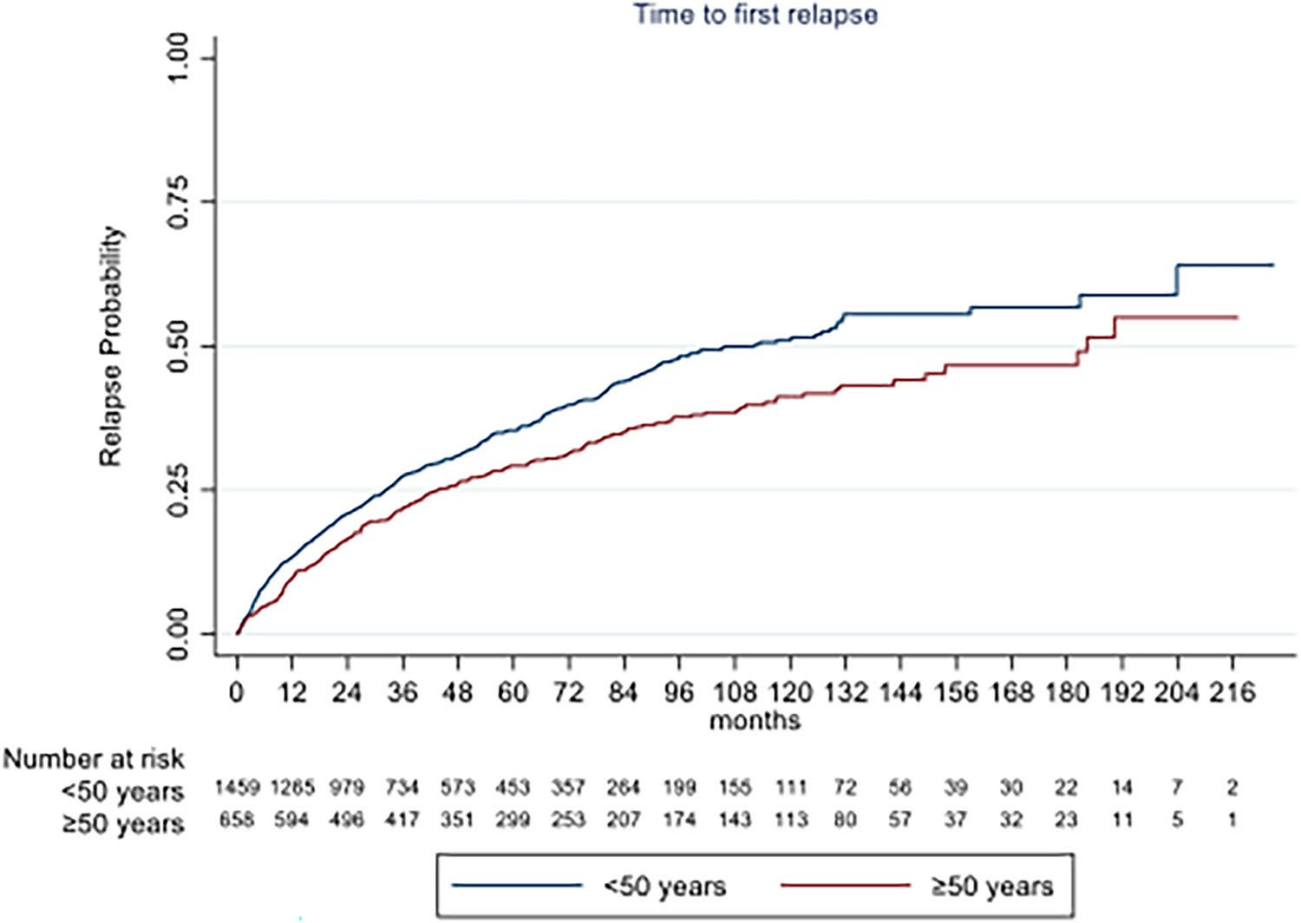
Cumulative probability for experiencing a relapse in RRMS patients comparing younger and older cohort

Cox regression analysis of time to first relapse yielded a hazard ratio (HR) of 1.34 (95% CI 1.12–1.60, p = 0.001), indicating a significantly higher relapse risk for the younger cohort (Fig. 1).

In younger patients, the mean EDSS score of 1.6 (SD: 1.3) remained stable over the treatment period of 6.0 years (SD: 3.9) (p = 0.101). In contrast, mean EDSS increased significantly in the older cohort, from 2.6 (SD: 1.5; median: 2.5) to 3.1 (SD: 1.9; median: 2.5; p < 0.001) over 7.8 years (SD: 4.5).

**Fig. 2:**
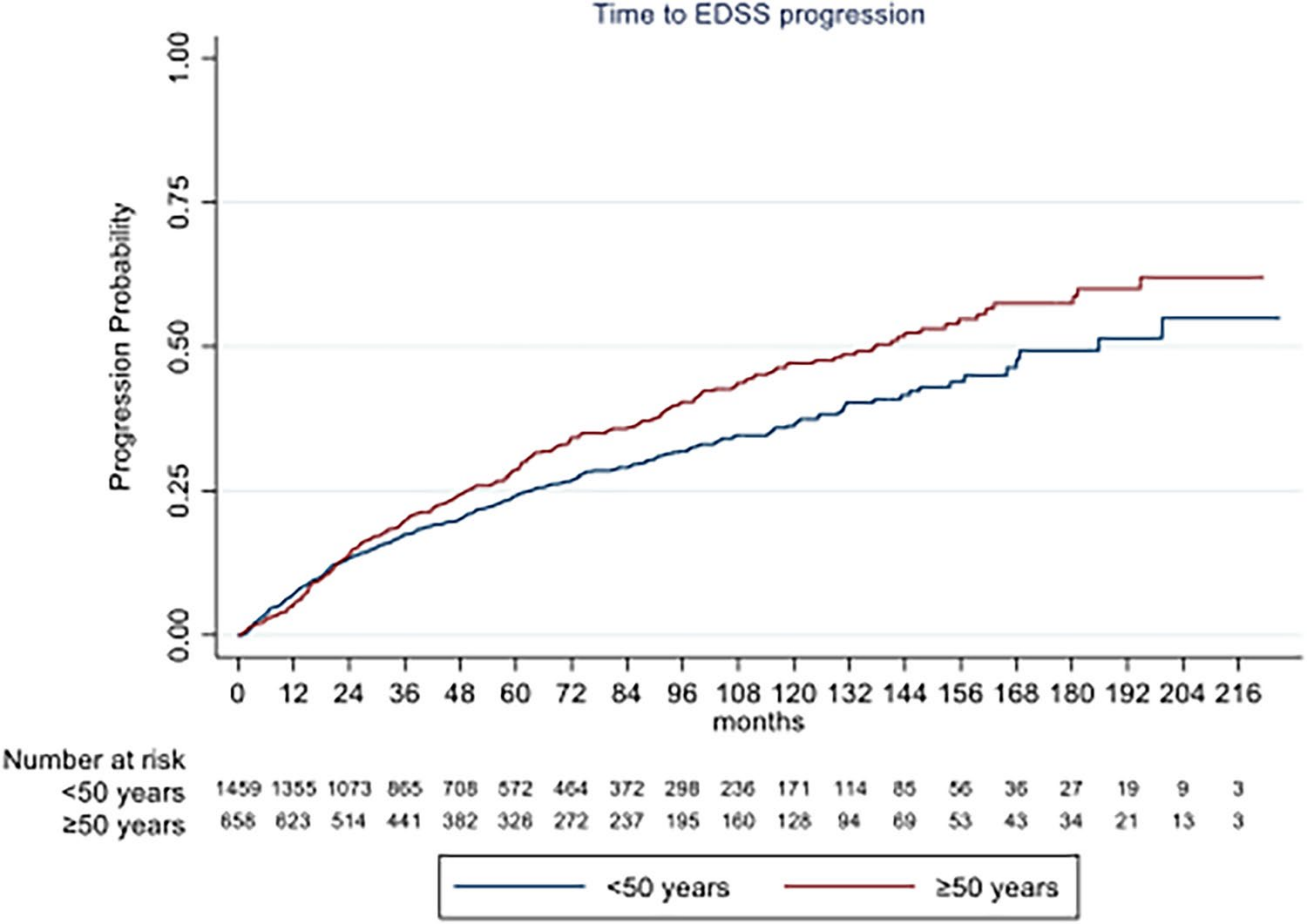
Cumulative probability for disability progression sustained for 12 weeks in RRMS patients comparing younger and older cohort

**Fig. 3:**
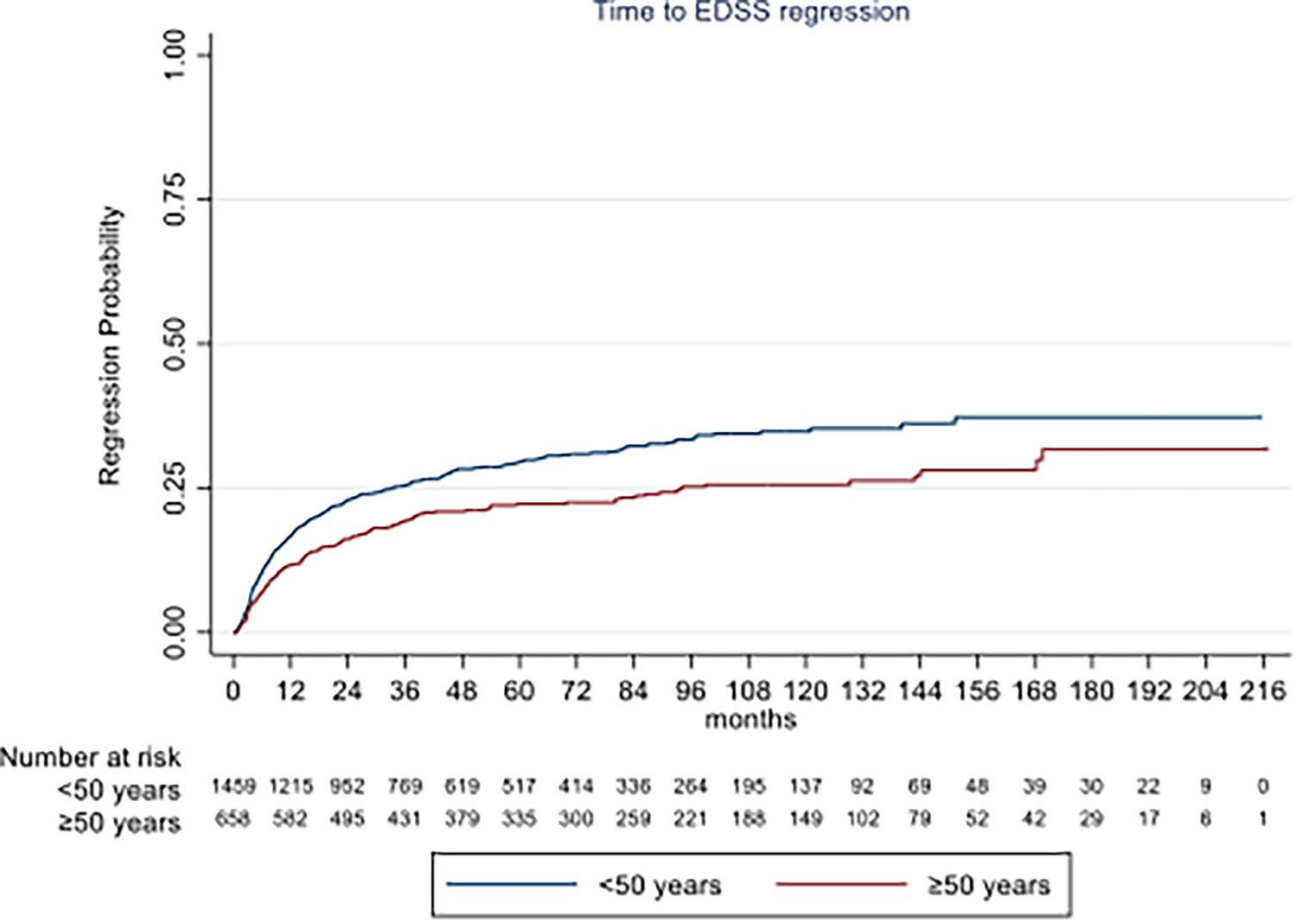
Cumulative probability for disability regression sustained for 12 weeks in RRMS patients comparing younger and older cohort

However, for sustained EDSS progression at 12 and 24 weeks, the corresponding HRs were 0.76 (95% CI 0.64–0.912, p = 0.003) and 0.70 (95% CI 0.58–0.85, p < 0.001), respectively, demonstrating a higher risk of disability progression in the older cohort (Fig. 2). In contrast, for sustained EDSS improvement at 12 and 24 weeks, the corresponding HRs were 1.49 (95% CI 1.21–1.82; p < 0.001) and 1.64 (95% CI 1.32–2.04; p < 0.001), respectively, indicating significantly greater EDSS improvement in the younger cohort (Fig. 3).

Factors significantly associated with a reduced relapse risk and a longer time to first relapse included male sex, lower baseline ARR, highly effective DMTs and shorter disease duration at baseline (prior to treatment initiation) as well as older age at the reference date (Table 3). In contrast, male sex, lower baseline EDSS scores and older age at the reference date were significantly associated with EDSS progression sustained for 12 and 24 weeks (Table 4).

**Table 3:** incidence rate ratio (IRR) of parameters predicting the endpoint annualized relapse rate

	IRR	95% Confidence Interval—lower	95% Confidence Interval—upper	Statistical significance (p-value)
Age group	1.039	0.871	1.241	0.670
Gender (female)	1.154	1.033	1.289	0.011
EDSS at baseline	1.016	0.974	1.059	0.458
Relapse rate within 12 months prior treatment start	1.264	1.201	1.324	< 0.001
Type of treatment (hDMT)	0.704	0.627	0.790	< 0.001
Duration of MS at baseline (years)	1.019	1.001	1.028	< 0.001
≥ 9 T2 lesions at abseline	1.045	0.898	1.217	0.566
≥ 1 Gd-enhancing T1 lesion at baseline	0.920	0.829	1.022	0.119
Age at reference date (years)	0.984	0.975	0.992	< 0.001

*EDSS* expanded disability status scale, *hDMT* highly effective DMT, *IRR* incidence rate ratio, Gd gadolinium, *MS* multiple sclerosis

**Table 4:** Hazard ratio (HR) of parameters predicting the endpoint EDSS progression sustained for 12 weeks

	HR	95% Wald Confidence Interval - lower	95% Wald Confidence Interval - upper	Statistical significance (p-value)
Age group	0.971	0.736	1.282	0.838
Gender (female)	0.793	0.672	0.935	0.006
EDSS at baseline	0.848	0.789	0.911	< 0.001
Relapse rate within 12 months prior treatment start	0.969	0.885	1.061	0.495
Type of treatment (hDMT)	1.014	0.846	1.216	0.880
Duration of MS at baseline (years)	1.011	0.997	1.025	0.116
≥ 9 T2 lesions at abseline	0.971	0.770	1.223	0.802
≥ 1 Gd-enhancing T1 lesion at baseline	0.886	0.751	1.047	0.155
Age at reference date (years)	1.016	1.002	1.030	0.024

*EDSS* expanded disability status scale, *hDMT* highly effective DMT, *IRR* incidence rate ratio, Gd gadolinium, *MS* multiple sclerosis

Conversely, a higher number of gadolinium-enhancing lesions, higher ARR and EDSS scores and shorter disease duration at baseline as well as younger age at the reference date were associated with sustained EDSS improvement at both 12 and 24 weeks (Table 5).

**Table 5:** Hazard ratio (HR) of parameters predicting the endpoint EDSS improvement sustained for 12 weeks

	HR	95% Wald Confidence Interval—lower	95% Wald Confidence Interval—upper	Statistical significance (p-value)
Age group	1.054	0.781	1.423	0.729
Gender (female)	1.036	0.868	1.237	0.696
EDSS at baseline	1.391	1.309	1.479	< 0.001
Relapse rate within 12 months prior treatment start	1.211	1.119	1.311	< 0.001
Type of treatment (hDMT)	0.950	0.784	1.152	0.602
Duration of MS at baseline (years)	0.959	0.943	0.975	< 0.001
≥ 9 T2 lesions at abseline	1.084	0.852	1.378	0.513
≥ 1 Gd-enhancing T1 lesion at baseline	1.247	1.048	1.484	0.013
Age at reference date (years)	0.979	0.965	0.992	0.002

*EDSS* expanded disability status scale, *hDMT* highly effective DMT, *IRR* incidence rate ratio, Gd gadolinium, *MS* multiple sclerosis

The most common reasons for treatment switching during the observation period included insufficient efficacy, particularly under DMF, FTY and TERI, a combination of JCV antibody seropositivity and NTZ treatment duration exceeding two years, patient preference and adverse events (AEs). It should be noted that treating neurologists were able to report multiple reasons per patient.

The most frequent adverse events in both age groups were related to disorders of the skin and subcutaneous tissue (n = 146, 10% vs. n = 56, 8.5%), gastrointestinal disorders (n = 94, 6.4% vs. n = 41, 6.2%), blood and lymphatic system disorders (n = 73, 5% vs. n = 53, 8.1%; p = 0.006), infections (n = 43, 2.9% vs. n = 34, 5.2%; p = 0.012) and infusion-related reactions (n = 38, 2.6% vs. n = 21, 3.2%). With the exception of blood and lymphatic system disorders and infections, the distribution of adverse events was comparable between the two age groups.

## Discussion

In this nationwide observational study conducted at well-defined MS treatment centers, we analyzed prospectively collected real-world data to evaluate the efficacy and safety of AZM, CLAD, DMF, FTY, NTZ, OCR, OFA, OZA, PONE, SIPO and TERI in patients with MS across different age groups.

As individuals with MS age, the benefits of highly effective DMTs may diminish, whereas low- or moderate-efficacy DMTs may continue to provide clinical benefit. This has been demonstrated in meta-analyses, which showed that high-efficacy DMTs were superior to moderate-efficacy DMTs only in patients younger than 54.2 and 40.5 years, respectively [30, 32]. In contrast, several studies investigating DMTs approved for active or highly active disease courses have reported comparable efficacy across different age groups [6, 7, 16].

However, abrupt discontinuation or prolonged treatment gaps between sequential DMTs—particularly therapies affecting lymphocyte trafficking or migration, such as NTZ and S1PR modulators—may lead to reoccurrence of inflammatory disease activity, even in patients older than 50 years [3, 5, 8, 15, 18, 21, 24].

An alternative strategy to mitigate the risks associated with treatment discontinuation is extended interval dosing (EID), which has demonstrated promising efficacy for both NTZ and OCR [1, 9, 28].

Baseline characteristics in our cohort were largely consistent with those reported in the literature, particularly with respect to EDSS scores in older patients [2, 6, 7, 16, 22].

Approximately one-third of treated MS patients in our registry were older than 50 years, which aligns with findings from other real-world studies [12, 18, 26].

We observed a significantly higher relapse risk—reflected by an ARR and time to first relapse—in the younger cohort across treatments used for mild, moderate, and highly active disease courses. This finding is consistent with a previous report [22], but contrasts with earlier results [6]. In addition, and in contrast to some prior studies, we demonstrated a higher risk of sustained EDSS progression in the older cohort [6, 16].

Adverse event rates were comparable between age groups in our cohort, with no evidence of an increased risk of opportunistic infections or malignancies, in line with findings from recent literature [2, 6, 7, 11, 16, 22].

A major strength of this study is the nationwide observational design with comprehensive data collection from certified MS clinics, encompassing Austrian patients treated with a broad range of DMTs, including AZM, CLAD, DMF, FTY, NTZ, OCR, OFA, OZA, PONE, SIPO, and TERI.

Nevertheless, some limitations of our study should also be considered. MRI data were available only at baseline prior to treatment initiation and were included as independent variables in the outcome models; longitudinal imaging data during follow-up were not available. Treatment selection may also have been influenced by individual clinical considerations guiding the treating neurologist’s decision. In Austria, treatment strategies follow expert statements for each therapy published on the AMSTR website, which are aligned with European MS treatment recommendations and incorporate national reimbursement regulations [23, 33].

Our study predominantly included patients who initiated treatment with DMF, NTZ and FTY. At about one third of patients switched treatment, most commonly to B-cell depleting therapies and CLAD. This pattern reflects current treatment recommendations favouring escalation to more effective therapies when indicated [23, 25, 33].

Furthermore, while the older cohort had a mean age of 56 years at the reference date, patients were on average 47 years old at diagnosis, which should be considered when interpreting analyses based on age categories.

Finally, due to our inclusion criterion requiring a minimum follow-up period of 12 months, approximately 16% of patients in the younger cohort and 8% in the older cohort were excluded because their treatment duration was shorter. Given the relatively small proportion of excluded patients in both cohorts, we do not believe that these missing data substantially affected the outcome measures. Nevertheless, as patients with higher disease activity are more likely to discontinue treatment earlier, excluding these patients may have led to an underestimation of ARR and progression rates in both the younger and older groups.

In summary, we observed a higher risk of relapses and greater EDSS improvement in the younger cohort, whereas the older cohort showed greater EDSS worsening. Additionally, adverse events were comparable between MS patients younger and older than 50 years across multiple DMTs. Relapse probability and EDSS progression and improvement were influenced by sex, baseline EDSS, ARR, gadolinium-enhancing lesions, disease duration, type of treatment and age at the reference date. These findings suggest that therapeutic efficacy is determined both by disease activity and by chronological age.

The substantial proportion of older patients in our cohort highlights the clinical relevance of treatment decisions in this population. Our results should be taken into account in individualized, shared treatment decisions for older MS patients, alongside considerations of immunosenescence and comorbidities.

## Data Availability

The data that support the findings of this study are available from the corresponding author upon reasonable request.

## References

[1] Baig MMA, Siddiqui FZ, Ashkar A, Naeem A, Ahmed S, Waqas SA (2025) Comparing the efficacy and safety of extended vs standard dosing of ocrelizumab in MS: a systemic review and meta-analysis. Mult Scler Relat Disord 94:106257. 10.1016/j.msard.2025.106257 39805179 10.1016/j.msard.2025.106257

[2] Berkovich R, Negroski D, Wynn D, Sellers D, Bzdek KG, Lublin AL, Rawlings AM, Quach C, Wells DP, Dumlao M, Bora A, Ranno AE, Luo KL, Chavin J, Hua LH, Becker D (2023) Effectiveness and safety of switching to teriflunomide in older patients with relapsing multiple sclerosis: a real-world retrospective multicenter analysis. Mult Scler Relat Disord 70:104472. 10.1016/j.msard.2022.104472 36566698 10.1016/j.msard.2022.104472

[3] Bsteh G, Introcaso V, Gradl C, Traxler G, Barket R, Föttinger F, Hammer HN, Krajnc N, Ponleitner M, Zrzavy T, Deisenhammer F, Pauli FD, Chan A, Berger T, Hoepner R, Hegen H (2025) Risk stratification for disease reactivation after therapy de-escalation/discontinuation in relapsing multiple sclerosis by the VIAADISC score. Mult Scler Relat Disord 103:106691. 10.1016/j.msard.2025.106691 40845598 10.1016/j.msard.2025.106691

[4] Chertcoff A, Ng HS, Zhu F, Zhao Y, Tremlett H (2023) Polypharmacy and multiple sclerosis: a population-based study. Mult Scler (Houndmills, Basingstoke, England) 29(1):107–118. 10.1177/13524585221122207 10.1177/13524585221122207 PMC9896267 36301629

[5] Corboy JR, Fox RJ, Kister I, Cutter GR, Morgan CJ, Seale R, Engebretson E, Gustafson T, Miller AE, DISCOMS investigators (2023) Risk of new disease activity in patients with multiple sclerosis who continue or discontinue disease-modifying therapies (DISCOMS): a multicentre, randomised, single-blind, phase 4, non-inferiority trial. Lancet Neurol 22(7):568–577. 10.1016/S1474-4422(23)00154-0 37353277 10.1016/S1474-4422(23)00154-0

[6] Disanto G, Moccia M, Sacco R, Spiezia AL, Carotenuto A, Brescia Morra V, Gobbi C, Zecca C (2022) Monitoring of safety and effectiveness of cladribine in multiple sclerosis patients over 50 years. Mult Scler Relat Disord 58:103490. 10.1016/j.msard.2022.103490 35007823 10.1016/j.msard.2022.103490

[7] Epstein S, Fong KT, De Jager PL, Levine L, Riley C, Wesley S, Vargas WS, Farber R (2021) Evaluation of ocrelizumab in older progressive multiple sclerosis patients. Mult Scler Relat Disord 55:103171. 10.1016/j.msard.2021.103171 34329872 10.1016/j.msard.2021.103171

[8] Fagius J, Feresiadou A, Larsson E-M, Burman J (2017) Discontinuation of disease modifying treatments in middle aged multiple sclerosis patients. First line drugs vs natalizumab. Mult Scler Relat Disord 12:82–87. 10.1016/j.msard.2017.01.009 28283113 10.1016/j.msard.2017.01.009

[9] Foley JF, Defer G, Ryerson LZ, Cohen JA, Arnold DL, Butzkueven H, Cutter G, Giovannoni G, Killestein J, Wiendl H, Smirnakis K, Xiao S, Kong G, Kuhelj R, Campbell N, van der Walt A, Dwyer C, Buzzard K, Spies J, Parratt J, van Pesch V, Willekens B, Perrotta G, Bartholomé E, Grand’Maison F, Jacques F, Giacomini P, Vosoughi R, Girard J-M, de Seze J, Lebrun Frenay C, Ruet A, Laplaud D-A, Reifschneider G, Wagner B, Rauer S, Pul R, Seipelt M, Berthele A, Klotz L, Kallmann B-A, Paul F, Achiron A, Lus G, Centonze D, Patti F, Grimaldi L, Hupperts R, Frequin S, Fermont J, Madueno SE, Alonso Torres AM, Costa-Frossard França L, Meca-Lallana JE, Ruiz LB, Pearson O, Rog D, Evangelou N, Ismail A, Lathi E, Fox E, Leist T, Sloane J, Wu G, Khatri B, Steingo B, Thrower B, Gudesblatt M, Calkwood J, Bandari D, Scagnelli C, Laganke C, Robertson D, Kipp L, Belkin M, Cohan S, Goldstick L, Courtney A, Vargas W, Sylvester A, Srinivasan J, Kannan M, Picone M, English J, Napoli S, Balabanov R, Zaydan I, Nicholas J, Kaplan J, Lublin F, Riser E, Miller T, Alvarez E, Wray S, Gross J, Pawate S, Hersh C, McCarthy L, Crayton H, Graves J (2022) Comparison of switching to 6-week dosing of natalizumab versus continuing with 4-week dosing in patients with relapsing-remitting multiple sclerosis (NOVA): a randomised, controlled, open-label, phase 3b trial. Lancet Neurol 21(7):608–619. 10.1016/S1474-4422(22)00143-0 35483387 10.1016/S1474-4422(22)00143-0

[10] Fox RJ, Bar-Or A, Traboulsee A, Oreja-Guevara C, Giovannoni G, Vermersch P, Syed S, Li Y, Vargas WS, Turner TJ, Wallstroem E, Reich DS, HERCULES Trial Group (2025) Tolebrutinib in nonrelapsing secondary progressive multiple sclerosis. N Engl J Med 392(19):1883–1892. 10.1056/NEJMoa2415988 40202696 10.1056/NEJMoa2415988

[11] Giovannoni G, Coyle PK, Vermersch P, Walker B, Aldridge J, Nolting A, Galazka A, Lemieux C, Leist TP (2021) Integrated lymphopenia analysis in younger and older patients with multiple sclerosis treated with cladribine tablets. Front Immunol 12:763433. 10.3389/fimmu.2021.763433 35003076 10.3389/fimmu.2021.763433 PMC8740297

[12] Goereci Y, Ellenberger D, Rommer P, Dunkl V, Golla H, Zettl U, Stahmann A, Warnke C (2024) Persons with multiple sclerosis older than 55 years: an analysis from the German MS registry. J Neurol 271(6):3409–3416. 10.1007/s00415-024-12286-4 38517521 10.1007/s00415-024-12286-4 PMC11136707

[13] Guger M, Enzinger C, Leutmezer F, Kraus J, Kalcher S, Kvas E, Berger T (2018) Real-life clinical use of natalizumab and fingolimod in Austria. Acta Neurol Scand 137(2):181–187. 10.1111/ane.12864 29159801 10.1111/ane.12864

[14] Guger M, Enzinger C, Leutmezer F, Kraus J, Kalcher S, Kvas E, Berger T, Austrian MS Treatment Registry (AMSTR) (2020) Oral therapies for treatment of relapsing-remitting multiple sclerosis in Austria: a 2-year comparison using an inverse probability weighting method. J Neurol 267(7):2090–2100. 10.1007/s00415-020-09811-6 32246251 10.1007/s00415-020-09811-6 PMC7320928

[15] Hatcher SE, Waubant E, Nourbakhsh B, Crabtree-Hartman E, Graves JS (2016) Rebound syndrome in patients with multiple sclerosis after cessation of fingolimod treatment. JAMA Neurol 73(7):790–794. 10.1001/jamaneurol.2016.0826 27135594 10.1001/jamaneurol.2016.0826

[16] Hua LH, Bar-Or A, Cohan SL, Lublin FD, Coyle PK, Cree BA, Meng X, Su W, Cox GM, Fox RJ (2023) Effects of baseline age and disease duration on the efficacy and safety of siponimod in patients with active SPMS: post hoc analyses from the EXPAND study. Mult Scler Relat Disord 75:104766. 10.1016/j.msard.2023.104766 37245350 10.1016/j.msard.2023.104766

[17] Iaquinto S, Chan A, Manjaly Z-M, Stanikić M, Ineichen BV, Kuhle J, Haag C, Müller J, Yaldizli Ö, Kamm CP, Calabrese P, Zecca C, Magnusson T, Ammann S, Kesselring J, Baum C, Kaminski M, Puhan MA, von Wyl V (2025) Rising prevalence of multiple sclerosis in Switzerland: results from the Swiss multiple sclerosis registry. Neuroepidemiology 59(6):623–632. 10.1159/000542632 39557017 10.1159/000542632 PMC12688380

[18] Jouvenot G, Courbon G, Lefort M, Rollot F, Casey R, Le Page E, Michel L, Edan G, de Seze J, Kremer L, Bigaut K, Vukusic S, Mathey G, Ciron J, Ruet A, Maillart E, Labauge P, Zephir H, Papeix C, Defer G, Lebrun-Frenay C, Moreau T, Laplaud DA, Berger E, Stankoff B, Clavelou P, Thouvenot E, Heinzlef O, Pelletier J, Al-Khedr A, Casez O, Bourre B, Cabre P, Wahab A, Magy L, Camdessanché J-P, Doghri I, Moulin S, Ben-Nasr H, Labeyrie C, Hankiewicz K, Neau J-P, Pottier C, Nifle C, Collongues N, Kerbrat A, Cotton F, Douek P, Guillememin F, Pachot A, Olaiz J, Rigaud-Bully C, Marignier R, Debouverie D, Lubetzki C, Cohen M, Fromont A, Wiertlewsky S, Audoin B, Giannesini C, Gout O, Montcuquet A, Bakchine S, Maurousset A, Maubeuge N, OFSEP Investigators (2024) High-efficacy therapy discontinuation vs continuation in patients 50 years and older with nonactive MS. JAMA Neurol 81(5):490–498. 10.1001/jamaneurol.2024.0395 38526462 10.1001/jamaneurol.2024.0395 PMC10964164

[19] Lee K-A, Flores RR, Jang IH, Saathoff A, Robbins PD (2022) Immune senescence, immunosenescence and aging. Front Aging 3:900028. 10.3389/fragi.2022.900028 35821850 10.3389/fragi.2022.900028 PMC9261375

[20] Macaron G, Larochelle C, Arbour N, Galmard M, Girard JM, Prat A, Duquette P (2023) Impact of aging on treatment considerations for multiple sclerosis patients. Front Neurol 14:1197212. 10.3389/fneur.2023.1197212 37483447 10.3389/fneur.2023.1197212 PMC10361071

[21] Malpas CB, Roos I, Sharmin S, Buzzard K, Skibina O, Butzkueven H, Kappos L, Patti F, Alroughani R, Horakova D, Havrdova EK, Izquierdo G, Eichau S, Hodgkinson S, Grammond P, Lechner-Scott J, Kalincik T, the MSBase Study Group (2022) Multiple sclerosis relapses following cessation of fingolimod. Clin Drug Investig 42(4):355–364. 10.1007/s40261-022-01129-7 35303292 10.1007/s40261-022-01129-7 PMC8989797

[22] Mao-Draayer Y, Bar-Or A, Balashov K, Foley J, Smoot K, Longbrake EE, Robertson D, Mendoza JP, Lewin JB, Everage N, Božin I, Lyons J, Mokliatchouk O, Bame E, Giuliani F (2025) Real-world safety and effectiveness of dimethyl fumarate in patients with MS: results from the ESTEEM Phase 4 and PROCLAIM Phase 3 studies with a focus on older patients. Adv Ther 42(1):395–412. 10.1007/s12325-024-03047-w 39570545 10.1007/s12325-024-03047-w PMC11782338

[23] Montalban X, Gold R, Thompson AJ, Otero-Romero S, Amato MP, Chandraratna D, Clanet M, Comi G, Derfuss T, Fazekas F, Hartung HP, Havrdova E, Hemmer B, Kappos L, Liblau R, Lubetzki C, Marcus E, Miller DH, Olsson T, Pilling S, Selmaj K, Siva A, Sorensen PS, Sormani MP, Thalheim C, Wiendl H, Zipp F (2018) ECTRIMS/EAN guideline on the pharmacological treatment of people with Multiple Sclerosis. Eur J Neurol 25(2):215–237. 10.1111/ene.13536 29352526 10.1111/ene.13536

[24] O’Connor PW, Goodman A, Kappos L, Lublin FD, Miller DH, Polman C, Rudick RA, Aschenbach W, Lucas N (2011) Disease activity return during natalizumab treatment interruption in patients with Multiple Sclerosis. Neurology 76(22):1858–1865. 10.1212/WNL.0b013e31821e7c8a 21543733 10.1212/WNL.0b013e31821e7c8a

[25] Oreja-Guevara C, Martínez-Yélamos S, Eichau S, Llaneza MÁ, Martín-Martínez J, Peña-Martínez J, Meca-Lallana V, Alonso-Torres AM, Moral-Torres E, Río J, Calles C, Ares-Luque A, Ramió-Torrentà L, Marzo-Sola ME, Prieto JM, Martínez-Ginés ML, Arroyo R, Otano-Martínez MÁ, Brieva-Ruiz L, Gómez-Gutiérrez M, Rodríguez-Antigüedad A, Galán Sánchez-Seco V, Costa-Frossard L, Hernández-Pérez MÁ, Landete-Pascual L, González-Platas M, Meca-Lallana JE (2024) Beyond lines of treatment: Embracing early high-efficacy disease-modifying treatments for Multiple Sclerosis management. Ther Adv Neurol Disord 17:17562864241284372. 10.1177/17562864241284372 39483817 10.1177/17562864241284372 PMC11526321

[26] Piedrabuena MA, Correale J, Fiol M, Marrodan M, Rojas JI, Alonso M, Pappolla A, Miguez J, Patrucco L, Cristiano E, Vrech C, Cohen L, Alonso R, Silva B, Luetic G, Deri N, Burgos M, Liwacki S, Piedrabuena R, Tkachuk V, Barboza A, Martinez A, Balbuena ME, Pinheiro AA, Nofal P, Lopez PA, Tavolini D, Leguizamon F, Hryb JP, Tizio S, Recchia L, Reich E, Contentti EC, Marcilla MP, Pagani F, Cabrera LM, Curbelo MC, Mainella C, Liguori NF, Coppola M, Pettinicchi JP, Carra A, Jose G, Nadur D, Bestoso S, Pestchanker C, Vazquez GD, Martinez CM, Ysrraelit MC (2024) Selection of disease modifying therapies in Multiple Sclerosis based on patient’s age and disease activity: Data from a nationwide registry. J Neurol Sci 461:123052. 10.1016/j.jns.2024.123052 38797140 10.1016/j.jns.2024.123052

[27] Salter A, Lancia S, Kowalec K, Fitzgerald KC, Marrie RA (2024) Investigating the prevalence of comorbidity in Multiple Sclerosis clinical trial populations. Neurology 102(5):e209135. 10.1212/WNL.0000000000209135 38350062 10.1212/WNL.0000000000209135 PMC11067694

[28] Selmaj K, Hartung H-P, Mycko MP, Selmaj I, Cross AH (2024) MS treatment de-escalation: Review and commentary. J Neurol 271(10):6426–6438. 10.1007/s00415-024-12584-x 39093335 10.1007/s00415-024-12584-x PMC11447123

[29] Silva B, Casales F, Donoso CB, Varela L, Heriz A, Gonzalez C, Míguez J, Alonso R (2024) Safety of high efficacy therapies in older people with Multiple Sclerosis: a real-world evidence study. Mult Scler Relat Disord 90:105830. 10.1016/j.msard.2024.105830 39216455 10.1016/j.msard.2024.105830

[30] Vollmer BL, Wolf AB, Sillau S, Corboy JR, Alvarez E (2022) Evolution of disease modifying therapy benefits and risks: An argument for de-escalation as a treatment paradigm for patients with Multiple Sclerosis. Front Neurol 12:799138. 10.3389/fneur.2021.799138 35145470 10.3389/fneur.2021.799138 PMC8821102

[31] Wallin MT, Culpepper WJ, Campbell JD, Nelson LM, Langer-Gould A, Marrie RA, Cutter GR, Kaye WE, Wagner L, Tremlett H, Buka SL, Dilokthornsakul P, Topol B, Chen LH, LaRocca NG, US Multiple Sclerosis Prevalence Workgroup (2019) The prevalence of MS in the United States: a population-based estimate using health claims data. Neurology 92(10):e1029–e1040. 10.1212/WNL.0000000000007035 30770430 10.1212/WNL.0000000000007035 PMC6442006

[32] Weideman AM, Tapia-Maltos MA, Johnson K, Greenwood M, Bielekova B (2017) Meta-analysis of the Age-Dependent Efficacy of Multiple Sclerosis Treatments. Front Neurol. 10.3389/fneur.2017.00577 29176956 10.3389/fneur.2017.00577 PMC5686062

[33] Wiendl H, Gold R, Berger T, Derfuss T, Linker R, Mäurer M, Aktas O, Baum K, Berghoff M, Bittner S, Chan A, Czaplinski A, Deisenhammer F, Di Pauli F, Du Pasquier R, Enzinger C, Fertl E, Gass A, Gehring K, Gobbi C, Goebels N, Guger M, Haghikia A, Hartung H-P, Heidenreich F, Hoffmann O, Kallmann B, Kleinschnitz C, Klotz L, Leussink VI, Leutmezer F, Limmroth V, Lünemann JD, Lutterotti A, Meuth SG, Meyding-Lamadé U, Platten M, Rieckmann P, Schmidt S, Tumani H, Weber F, Weber MS, Zettl UK, Ziemssen T, Zipp F, Multiple Sclerosis Therapy Consensus Group (MSTCG) (2021) Multiple Sclerosis Therapy Consensus Group (MSTCG): position statement on disease-modifying therapies for Multiple Sclerosis (white paper). Ther Adv Neurol Disord 14:17562864211039648. 10.1177/17562864211039648 34422112 10.1177/17562864211039648 PMC8377320

[34] Zinganell A, Göbel G, Berek K, Hofer B, Asenbaum-Nan S, Barang M, Böck K, Bsteh C, Bsteh G, Eger S, Eggers C, Fertl E, Joldic D, Khalil M, Langenscheidt D, Komposch M, Kornek B, Kraus J, Krendl R, Rauschka H, Sellner J, Auer M, Hegen H, Pauli FD, Deisenhammer F (2024) Multiple sclerosis in the elderly: a retrospective cohort study. J Neurol 271(2):674–687. 10.1007/s00415-023-12041-1 37855871 10.1007/s00415-023-12041-1

